# Stem cells from umbilical cord blood do have myogenic potential, with and without differentiation induction *in vitro*

**DOI:** 10.1186/1479-5876-7-6

**Published:** 2009-01-14

**Authors:** Tatiana Jazedje, Mariane Secco, Natássia M Vieira, Eder Zucconi, Thomaz R Gollop, Mariz Vainzof, Mayana Zatz

**Affiliations:** 1 Department of Biology, Human Genome Research Center, São Paulo, Brazil; 2 Fetal Medicine Institute of São Paulo, São Paulo, Brazil

## Abstract

The dystrophin gene, located at Xp21, codifies dystrophin, which is part of a protein complex responsible for the membrane stability of muscle cells. Its absence on muscle causes Duchenne Muscular Dystrophy (DMD), a severe disorder, while a defect of muscle dystrophin causes Becker Muscular Dystrophy (DMB), a milder disease. The replacement of the defective muscle through stem cells transplantation is a possible future treatment for these patients. Our objective was to analyze the potential of CD34+ stem cells from umbilical cord blood to differentiate in muscle cells and express dystrophin, *in vitro*. Protein expression was analyzed by Immunofluorescence, Western Blotting (WB) and Reverse Transcriptase – Polymerase Chain Reaction (RT-PCR). CD34+ stem cells and myoblasts from a DMD affected patient started to fuse with muscle cells immediately after co-cultures establishment. Differentiation in mature myotubes was observed after 15 days and dystrophin-positive regions were detected through Immunofluorescence analysis. However, WB or RT-PCR analysis did not detect the presence of normal dystrophin in co-cultures of CD34+ and DMD or DMB affected patients' muscle cells. In contrast, some CD34+ stem cells differentiated in dystrophin producers' muscle cells, what was observed by WB, reinforcing that this progenitor cell has the potential to originate muscle dystrophin *in vitro*, and not just *in vivo *like reported before.

## Background

More than 30 different types of muscular dystrophies have been identified to date, ranging from adult forms with a mild course to severe childhood forms with a rapid progression. Among them, the most severe form, X-linked Duchenne Muscular Dystrophy (DMD), affects 1:3500 living boys. It's caused by a mutation in the dystrophin gene, leading to the absence of its product, dystrophin. Its allelic milder form, Becker Muscular Dystrophy (BMD) is 10 times less frequent than DMD [1-3]. It differs from the first form because patients have some functional dystrophin in their muscle, which may be defective in quantity and/or size. Both disorders are characterized by a progressive degeneration of the skeletal muscle. In DMD, affected boys are confined to a wheelchair around age 10–12 and without assisted ventilation death occurs usually before age 20 of cardiac arrest or respiratory failure. In BMD, the course is highly variable. Some patients are confined to a wheelchair before age 20 while other may remain ambulant beyond age 60 depending on how the gene mutation affects the dystrophin amount and or function [4-6].

The dystrophin gene, with 2.4 Mb and 79 exons is the largest human gene. Its product, the protein dystrophin has 427 kDa [7-9]. Dystrophin belongs to a complex of proteins (dystrophin-glycoprotein complex) responsible for the membrane maintenance of muscle cells. A primary deficiency in any of these proteins induces to a secondary deficient of the entire complex, causing different types of muscular dystrophies [10,11].

Many different therapies have been tested in DMD animal models and patients. A promising approach to the treatment of DMD is to restore dystrophin expression by repairing the defective muscle through cell therapy. Previous studies have suggested that hematopoietic stem cells can contribute to skeletal muscle regeneration. In normal and *mdx *mice (murine model of DMD), bone marrow (BM)-derived cells were shown to participate in skeletal muscle repair after induced damage [12-14]. However, the clinical usefulness of hematopoietic cell transplantation for muscular dystrophies such as DMD [15] depends on the expansion, homing and myogenic differentiation of transplanted cells.

In past decades, human umbilical cord blood (HUCB) has been explored as an alternative source to BM for cell transplantation and therapy because of its hematopoietic and nonhematopoietic (mesenchymal) components [16]. In contrast to bone marrow aspiration, HUCB is obtained by a simple, safe and painless procedure after birth.

Regarding myogenic potential, recent studies have shown that subpopulations of HUCB cells can differentiate into muscle cells [17,18]. Additionally, CD34, transmembrane glycophosphoprotein known to be expressed by human hematopoietic progenitor cells has recently been associated with both the quiescent and activated states of myogenic progenitor cells. [19]. More recently, the *in vivo *myogenic differentiation of human umbilical cord blood was observed after the injection into the *sjl *dystrophic mice, suggesting that human umbilical cord blood has myogenic precursors [20].

Although the positive results of the *in vivo *injections, the interaction of these cells with human dystrophic muscle cells is still unknown. Here we have investigated, for the first time, the potential of umbilical cord blood CD34+ stem cells to interact and differentiate into muscle cells when in direct contact with human DMD/DMB myoblasts, and their potential to restore the absent protein. Our results show CD34+ cells are able to participate in the myotube formation, resulting in the restoration of dystrophin expression. These findings represent a possible tool for future cell therapy applications in DMD disease and for other muscular dystrophies.

## Materials and methods

### Isolation and characterization of human CD34+ cells from the umbilical cord blood

CD34+ stem cells from human umbilical cord were obtained from healthy babies, born in Hospital Albert Einstein, in São Paulo, Brazil. All studies were approved by the ethical committee and were done after written consent. The cord blood was processed as described in the SuperMACSII manual (Miltenyi Biotec, Bergisch Gladbach, Germany) and the CD34+ stem cells were obtained by magnetic cell sorting, using the "CD34 progenitor cell isolation kit" (Miltenyi Biotec, Bergisch Gladbach, Germany).

The purity of CD34+ cells was determined for flow cytometry. Firstly, the immunomagnetically selected cells were incubated with the conjugated antibody anti-CD34-PerCP (BD Biosciences), in phosphate-buffered saline (PBS) at 4°C for 30 minutes, as recommended by the manufacturer. A total of 10,000 labeled cells were analyzed using Guava EasyCyte flow cytometer running Guava ExpressPlus software (Guava Technologies). The percentage of CD34+ cells present in the sample was assessed after correction for the percentage of cells reactive with the isotype control.

### Cell cultures

CD34+ cells were cultured and expanded into 25 cm^2 ^plastic culture flasks (Corning, New York, USA), in 5 mL with StemSpan SFEM (Serum Free Expansion-Medium) and with the cytokine cocktail CC100* (Stem Cell Technologie, British Columbia, Canada), which contains 100 ng/mL rh Flt-3 Ligand, 100 ng/mL rh Stem Cell Factor, 20 ng/mL rh IL-3 and 20 ng/mL rh IL-6. Medium was replaced once a week, by centrifugation at 1,400 rpm, for 5 minutes. Cells were kept in an incubator at 37°C and 5% CO_2_.

Myoblasts from 3 DMD and 2 DMB affected patients were obtained from biceps biopsies. They were implanted into 25 cm^2 ^plastic culture flasks (Corning, New York, USA) with 5 mL of Dubecco's Modified Medium (DMEM) high glucose, 20% Fetal Bovine Serum (FBS; Gibco/Invitrogen, California, USA), 100 U/mL of penicillin and 100 mg/mL of streptomicyn (Sigma-Aldrich, Missouri, USA) and amphotericin B (Cultilab, São Paulo, Brazil), and kept in an incubator at 37°C and 5% CO_2_.

In a ratio 3:1 (3 fold CD34+ stem cells: 1 fold DMD/DMB muscle cells), co-cultures were performed with 50% of the medium used for CD34+ stem cells and 50% of the medium used for myoblasts. They were established into 25 cm^2 ^plastic culture flasks (Corning, New York, USA) with 5 mL of medium or into a 10 cm^2 ^tissue culture chamber (Nunc, Illinois, USA), with 4 mL of medium. Co-cultures were kept in an incubator at 37°C and 5% CO_2 _until final analysis.

### Dystrophin Immunofluorescence (IF) and Western Blotting (WB)

Immunolabelling was performed as previous described [21] and cells were analyzed with an inverted microscope (Carl Zeiss, Jena, Germany). For WB analysis, myoblasts of a DMB affected patient, normal muscle cells and co-cultures were trypsinized by standard procedures, washed with PBS 1× and centrifuged for 7 minutes at 1,400 rpm. CD34+ cells were washed and centrifuged with PBS 1× for 7 minutes at 1,400 rpm. Cell pellets were transferred to 1,5 mL eppendorfs and processed as previously described [22]. In both methodologies monoclonal antibodies C and/or N-terminal anti-human dystrophin were used (kindly provided by the late Dr. L. V. B. Anderson).

### Bisbenzimide H33342 immunofluorescence of living cells

CD34+ stem cells nuclei were dyed with Bisbenzimide H33342, 5 μg/mL (Sigma-Aldrich, Missouri, USA) for 90 minutes in CO_2 _incubator, at 37°C, in the dark. After that, cells were washed in PBS 1× and cultured protected from light. Stained stem cells were used in co-cultures of DMD muscle cells and normal CD34+ stem cells from umbilical cord blood.

### Reverse Transcription – Polymerase Chain Reaction (RT-PCR)

Total RNA from myoblasts of a DMD affected patient (with deletion of exons 3–17), normal muscle cells, CD34 positive stem cells and co-cultures were obtained as previously described [23]. RNA concentration and purity were determined spectrophotometrically. RT-PCRs reactions were performed as recommended in the supplier's protocol of the kit "SuperScript One-Step RT-PCR with Platinum Taq" (Gibco/Invitrogen, California, USA). The dystrophin primers sequences for the amplification of exons 8, 12, 13 and 51, are available at Leiden website . RT-PCRs were performed with Perkin-Elmer thermal cycler (PE Applied Biosystems, California, USA) using conditions recommended by the supplier's protocol. The annealing temperature used was 60°C.

## Results

### Identification and characterization of CD34+ cells derived from blood

**Figure 1:**
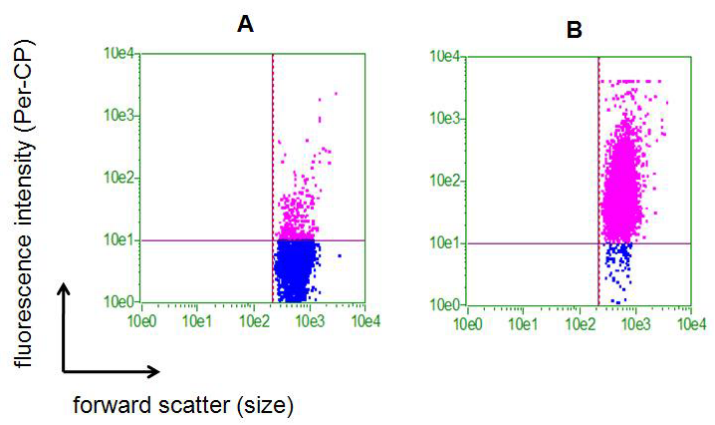
**CD34 flow cytometry**. Graphs show forward scatter versus fluorescence intensity. **a) **Unmarked control before CD34 purification with MACS columns, where 1.8% were CD34+. **b) **After CD34 purification with MACS columns, where 80.92% were CD34+. CD34+ cells are represented by pink points and CD34- cells are represented by blue points.

Cells isolated from human umbilical cord blood were immunomagnetically selected and characterized by flow cytometry. A representative subpopulation of the cells was CD34 positive (80.92%), as represented in the graphs (Figure 1).

### Cells co-cultures

**Figure 2:**
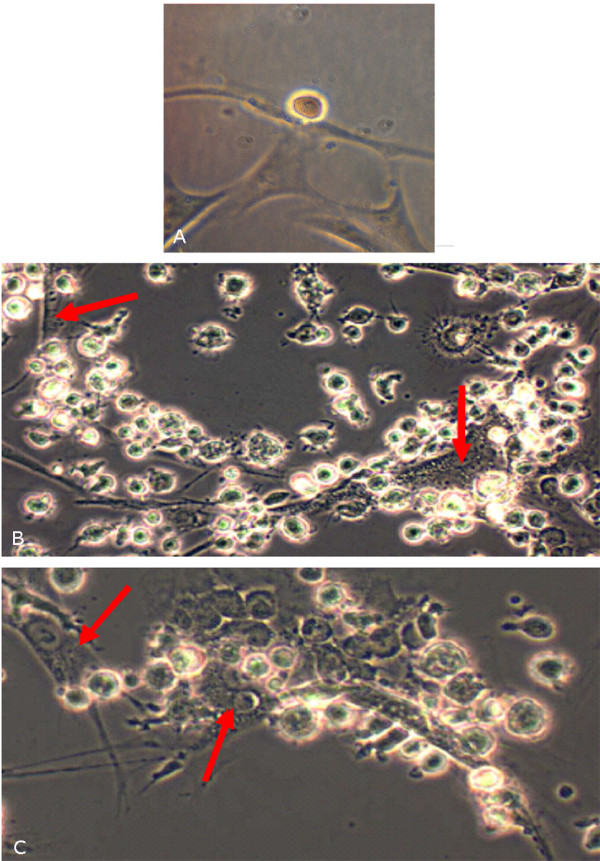
**Interaction between CD34+ stem cells and DMD myoblasts**. **a) **after 1 hour (630×); **b **and **c) **after 24 hours (200×). arrow indicate syncytium. Microscope Zeiss Axiovert 200.

**Figure 3:**
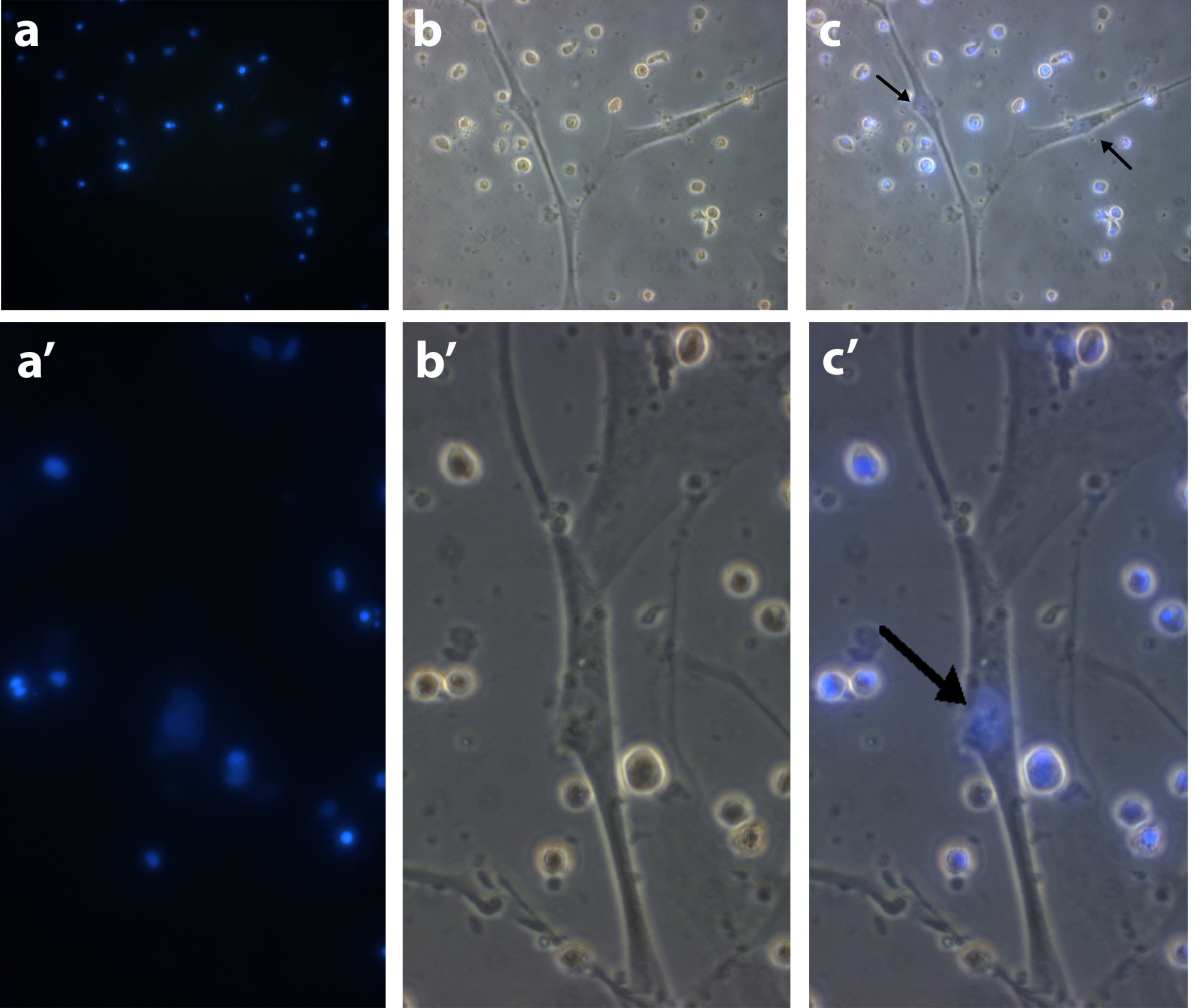
**Co-culture after 48 hours**. Before the co-culture, stem cell nuclei were previously stained with Bisbenzimide H33342 (blue fluorescence). **a) **CD34+ stem cell nuclei with blue fluorescence, been (a) 200× and (a') 630×, respectively. **b) **Halogen light of the co-culture, showing the co-existence of both cells: fluctuant CD34+ stem cells and adherent myoblasts, been (b) 200× and (b') 630×, respectively. **c) **Pictures from panels **a **and **b **superposed, showing blue nuclei inside adherent cells (black arrows), been (c) 200× and (c') 630×, respectively. Microscope Zeiss Axiovert 200.

Right after the co-culture establishment, the interaction between CD34+ and DMD myoblasts was observed (Figure 2). F, even that blue CD34+ nuclei were found inside the formed myotubes (Figure 3) the contact between the cells can ate the fusion, forming multinucleated syncytium. CD34+ stem cells and muscle cells division was also observed (data not shown).

### Dystrophin IF

**Figure 4:**
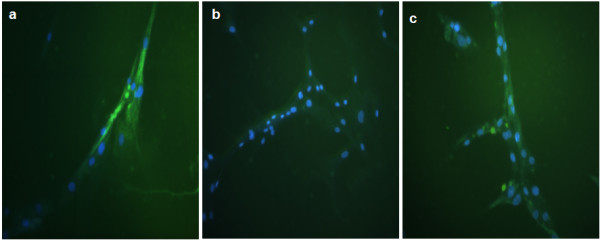
**Dystrophin IF in culture cells**. Anti-human dystrophin (N-terminal) FITC conjugated (green fluorescence) and nuclei dyed with Bisbenzimide H33342 (blue fluorescence). **a) **normal muscle cells, 200×; **b) **muscle cells of patient affected by DMD (dystrophin absent), 200×; **c) **Co-culture of stem cells CD34+ and muscle cells of patient affected by DMD, 200×. Microscope Zeiss Axiovert 200.

IF assay was performed after 15 days in culture. Co-cultures of CD34+ stem cells and DMD myoblasts showed positive dystrophin when compared with the normal myoblast culture (figure 4). This result suggests that the fusion of stem cells and muscle cells was sufficient to induce the stem cells nuclei to express muscle cells proteins, restoring the absent dystrophin expression. More than 3 different co-cultures of each patient, with different CD34+ cord blood stem cells donors, were analyzed. The same result were seen in relation to fusion and IF pattern.

**Figure 5:**
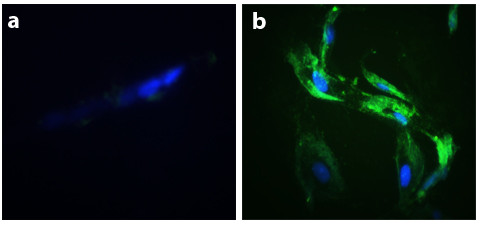
**Dystrophin IF after 15 days in culture**. Antibody anti-dystrophin N-terminal in green fluorescence. **a) **DMD muscle cells, after 15 days in culture, with nucleus dyed with Bisbenzimide H33342 (negative control); **b) **Co-culture after 15 days showing dystrophin expression and only the CD34+ stem cells' nuclei dyed with Bisbenzimide H33342. Microscope Zeiss Axiovert 200, 400×.

In addition to dystrophin IF analysis, the fusion of CD34+ stem cells and myoblasts from a DMD affected patient was also followed during the 15 days of culture through Bisbenzimide H33342 stem cells nuclei staining (figure 5).

### Western Blotting (WB) and RT-PCR analysis

We also evaluate the dystrophin expression by WB analysis. We did not detect the presence of normal dystrophin, by this method, after 15 days of co-cultures with CD34+ stem cells and DMB affected patient muscle cells (data not shown).

In order to confirm if there was any expression of dystrophin from the CD34+ stem cells, we used muscle cells from a DMD affected patient with deletion of exons 3–17 and total absence of dystrophin. Primers to amplify the exon 8 (inside the mutation) and exon 51 as a control were used. However, exon 8 was not amplified in co-cultures, indicating the absence or very low expression of dystrophin in co-cultures (data not shown).

### Transdifferentiation of CD34+ stem cells into muscle cells

During the expansion of CD34+ stem cells from umbilical cord blood, we observed the presence, in some cultures, of a small number of cells that became adherents. These cells were then kept in culture for 20 days with the same medium used in co-cultures (50% StemSpan CC100 and 50% DMEM). In this experiment, the used medium was filtered in a 0,22 μm filter (Millipore, Massachusetts, EUA) and the pH was adjusted to 7,4 with Hepes and Sodium Bicarbonate (Sigma-Aldrich, Missouri, USA).

**Figure 6:**
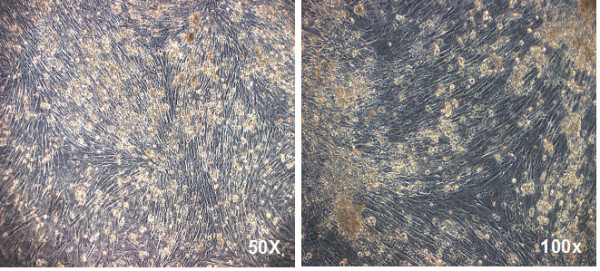
**CD34+ stem cells transdifferentiated in muscle cells *in vitro***. CD34+ stem cells that transdifferentiated in dystrophin producer muscle cells after 20 days in culture. Microscope Zeiss Axiovert 200.

**Figure 7:**
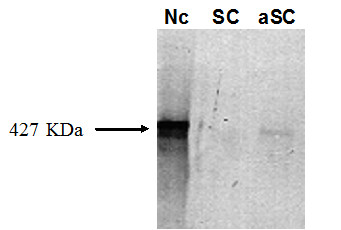
**Western blot for dystrophin**. WB analysis for dystrophin expression, after transdifferentiation of adherent cells obtained from prior CD34+ stem cells. **Nc) **normal control (human skeletal muscle). **SC) **CD34+ stem cells. **aSC) **adherent stem cells (prior CD34+).

A small number of adherent cells acquired the phenotype of differentiated muscle cells. At the 20^th ^day, a protein extract of these cells was analyzed by WB and the presence of normal dystrophin was observed (figures 6 and 7).

## Discussion

The possibility to replace a defective tissue by a normal one through stem cells transplantation has been proposed as an therapeutic approach for many disorders including muscular dystrophies. However, many experiments *in vitro *and *in vivo *will have to take place before an effective treatment for patients affected by muscular dystrophies will be available. Therefore, the understanding of stem cell biology is fundamental for their future utilization for therapeutic purposes.

The experiments showed here, demonstrated that the hematopoietic stem cells from umbilical cord blood have the potential to fuse to DMD muscle cells, restoring their dystrophin expression. However, co-culture experiments showed dystrophin expression only by IF analysis, suggesting a low expression oh this protein in co-cultured cells. On the other hand, IF is a much more sensitive method than WB, which also shows a greater variability.

Previous studies have suggested that hematopoietic stem cells can contribute to skeletal muscle regeneration. [16,20,24,25]. The report of a DMD patient who received a bone marrow (BM) transplantation from his father, at age 1, due to a severe combined immunodeficiency and who showed a mild course at age 14 [26] seems very promising. The presence of BM-derived donor nuclei in the muscle of this patient, suggested that exogenous hematopoietic human BM cells had the ability to fuse into recipient skeletal muscle and to persist for at least 13 years. However, these results have been questioned since the transplanted patient presents a high level of 44 and 45 exon skiping, leading to the production of an in-frame transcript, which could be responsible for his milder phenotype.

Cell fusion seems to be a rare phenomenon either *in vivo *or *in vitro *(1/100000 cells) and probably occurs in cell types where polyploidy is common, like hepatocytes, cardiac and skeletal muscle or purkinje cells. On the other hand, transdifferentiation is a process where the nuclei of the stem cells are reprogrammed, acquiring new expressed genes and proteins [27]. It was also observed that both endothelial progenitors in the embryo and differentiated endothelial cells from the umbilical vein transdifferentiated into beating cardiomyocytes, by fusion, when cocultured with neonatal rat cardiomyocytes or when injected near to a damaged area of the heart [28]. Transdifferentiation also occurred when murine bone marrow stem cells fused to murine embryonic stem cells [29]. However, the real meaning of fusion versus transdifferentiation is still controversial [30-33].

Adult stem cells transplantation in animal models also has shown controversial results [13,27,34]. In an attempt to follow the fate of exogenous stem cells *in vivo*, specific markers expression in transplanted stem cells, like GFP (Green Fluorescent Protein) or β-galactosidase are being used. However, green autofluorescent artifacts were observed in IF muscle analysis after stem cells transplantation in murines [35], calling the attention for the difficulty in the interpretation of published reports as well as on our own IF results.

Moreover, in most cases, it was not possible to compare results because of the differences of conditions in each experiment, such as the phenotype characterization and quantity of transplanted stem cells as well as the degeneration degree of the recipient musculature. Besides that, the microenvironmental conditions, presents *in vitro *or *in vivo *experiments are crucial to define and better understand the observed responses. Until very recently, our group showed that stem cells from HUCB did not differentiate into myotubes or express dystrophin when cultured in muscle-conditioned medium and in the presence of human muscle cells [25]. Subsequently wehuman Adipose Stem Cells (hASC) can participate in myotube formation when cultured with differentiating human DMD myoblasts and myotubes even when the co-culture was maintained in growth media [36]. The present results of co-culture of CD34+ and DMD myoblasts without the inductive media show that these cells can interact and express dystrophin. This data together with our previous findings [25] suggest that HUCB loose the capacity to fuse with muscle cells when they are previously committed. In other words, their pre-differentiation into muscle may alter or decrease their potential to fuse with muscle cells.

Probably, undifferentiated stem cells can respond to chemical factors released by the DMD muscle, providing the signals that contribute to the establishment of a favorable microenvironment to initiate the fusion and myogenic differentiation process. Others have also demonstrated that signals from damaged but not undamaged skeletal muscle induce myogenic differentiation of rat bone-marrow-derived mesenchymal stem cells [37]. Although a comprehensive analysis of the component(s) responsible for the myogenic effects has not been performed, we do not exclude the possibility that inflammatory and growth factors with myogenic effect, like IL6/LIF, IGF, HGF, or others [38-40] are present in the medium and are involved in the reported effects on human stem cells. Based on our experience, the IGF-1 concentration was significantly higher in the dystrophic muscle-conditioned medium than normal muscle medium (unpublished data).

Our results on WB analysis confirm the potential of umbilical cord blood CD34+ stem cells to differentiate in muscle cells *in vitro*, although the observed expression of dystrophin would not be enough for therapeutic potential. In fact, the skeletal myogenesis is a developmental cascade controlled by a family of myogenic regulatory factors, that are expressed with a well-defined time course, during the early stage of myogenic differentiation. Dystrophin is one of the last muscle proteins produced at the time of cell fusion [41]. So, it is possible that once differentiation is triggered, the expression of the genetic repertoire of a differentiated tissue *in vivo *may differ from the observed *in vitro*.

## Conclusion

Our findings showed that umbilical cord blood CD34+ stem cells have the potential to interact with dystrophic muscle cells restoring the dystrophin expression of DMD cells *in vitro*. Although utilized within the context of DMD, the results presented here may be valid to other muscle-related cell therapy applications.

## Competing interests

The authors declare that they have no competing interests.

## Authors' contributions

TJ and MZ conceived the study and wrote the manuscript. TJ designed and performed tissue culture, Western Blotting and Immunofluorescence. MS, NMV and EZ helped with flow cytometric evaluation and with the manuscript review. MV helped with Western Blotting and Immunofluorescence interpretation. TRG helped providing umbilical cord blood.

## References

[B1] Emery AEH (1993). Duchenne muscular dystrophy.

[B2] Emery AE (2002). The muscular dystrophies. Lancet.

[B3] Emery AE (2002). Muscular dystrophy into the new millennium. Neuromuscul Disord.

[B4] Passos-Bueno MR, Vainzof M, Marie SK, Zatz M (1994). Half the dystrophin gene apparently enough for a mild clinical course: confirmation of its potential use for gene therapy. Hum Mol Genet.

[B5] McNally EM, Passos-Bueno MR, Bönnemann CG, Vainzof M, de Sá Moreira E, Lidov HG, Othmane KB, Denton PH, Vance JM, Zatz M, Kunkel LM (1996). Mild and severe muscular dystrophy caused by a single γ-sarcoglycan mutation. Am J Hum Genet.

[B6] Bönnemann CG, Passos-Bueno MR, McNally EM, Vainzof M, de Sá Moreira E, Marie SK, Pavanello RC, Noguchi S, Ozawa E, Zatz M, Kunkel LM (1996). Genomic screening for β-sarcoglycan gene mutations: missense mutations may cause severe limb-girdle muscular dystrophy type 2E (LGMD 2E). Hum Mol Gen.

[B7] Koenig M, Hoffman EP, Bertelson CJ, Monaco AP, Feener C, Kunkel LM (1987). Complete cloning of the Duchenne muscular dystrophy (DMD) cDNA and preliminary genomic organization of the DMD gene in normal and affected individuals. Cell.

[B8] Zubrzycka-Gaarn EE, Bulman DE, Karpati G, Burghes AH, Belfall B, Klamut HJ, Talbot J, Hodges RS, Ray PN, Worton RG (1988). The Duchenne muscular dystrophy gene product is localized in sarcolemma of human skeletal muscle. Nature.

[B9] Arahata K, Ishiura S, Ishiguro T, Tsukahara T, Suhara Y, Eguchi C, Ishihara T, Nonaka I, Ozawa E, Sugita H (1988). Immunostaining of skeletal and cardiac muscle surface membrane with antibody against Duchenne muscular dystrophy peptide. Nature.

[B10] Ervasti JM, Campbell KP (1991). Membrane organization of the dystrophin-glycoprotein complex. Cell.

[B11] Vainzof M, Passos-Bueno MR, Canovas M, Moreira ES, Pavanello RC, Marie SK, Anderson LV, Bonnemann CG, McNally EM, Nigro V, Kunkel LM, Zatz M (1996). The sarcoglycan complex in the six autosomal recessive limb-girdle muscular dystrophies. Hum Mol Genet.

[B12] Ferrari G, Cusella-De Angelis G, Coletta M, Paolucci E, Stornaiuolo A, Cossu G, Mavilio F (1998). Muscle regeneration by bone marrow-derived myogenic progenitors. Science.

[B13] Gussoni E, Soneoka Y, Strickland CD, Buzney EA, Khan MK, Flint AF, Kunkel LM, Mulligan RC (1999). Dystrophin expression in the mdx mouse restored by stem cell transplantation. Nature.

[B14] Corbel SY, Lee A, Yi L, Duenas J, Brazelton TR, Blau HM, Rossi FM (2003). Contribution of hematopoietic stem cells to skeletal muscle. Nat Med.

[B15] Cossu G, Sampaolesi M (2004). New therapies for muscular dystrophy: cautious optimism. Trends Mol Med.

[B16] Erices A, Conget P, Minguell JJ (2000). Mesenchymal progenitor cells in human umbilical cord blood. Br J Haematol.

[B17] Ishikawa H, Nakao K, Matsumoto K, Ichikawa T, Hamasaki K, Nakata K, Eguchi K (2003). Antiangiogenic gene therapy for hepatocellular carcinoma using angiostatin gene. Hepatology.

[B18] Pesce M, Orlandi A, Iachininoto MG, Straino S, Torella AR, Rizzuti V, Pompilio G, Bonanno G, Scambia G, Capogrossi MC (2003). Myoendothelial differentiation of human umbilical cord blood-derived stem cells in ischemic limb tissues. Circ Res.

[B19] Beauchamp NJ, van Achterberg TA, Engelse MA, Pannekoek H, de Vries CJ (2003). Gene expression profiling of resting and activated vascular smooth muscle cells by serial analysis of gene expression and clustering analysis. Genomics.

[B20] Kong KY, Ren J, Kraus M, Finklestein SP, Brown RH (2004). Human umbilical cord blood cells differentiate into muscle in sjl muscular dystrophy mice. Stem Cells.

[B21] Deval E, Levitsky DO, Marchand E, Cantereau A, Raymond G, Cognard C (2002). Na(+)/Ca(2+) exchange in human myotubes: intracellular calcium rises in response to external sodium depletion are enhanced in DMD. Neuromuscul Disord.

[B22] Sunada Y, Edgar TS, Lotz BP, Rust RS, Campbell KP (1995). Merosin-negative congenital muscular dystrophy associated with extensive brain abnormalities. Neurology.

[B23] Chomczynski P, Sacchi N (1987). Single-step method of RNA isolation by acid guanidinium thiocyanate-phenol-chloroform extraction. Anal Biochem.

[B24] Gang EJ, Jeong JA, Hong SH, Hwang SH, Kim SW, Yang IH, Ahn C, Han H, Kim H (2004). Skeletal myogenic differentiation of mesenchymal stem cells isolated from human umbilical cord blood. Stem Cells.

[B25] Nunes VA, Cavaçana N, Canovas M, Strauss BE, Zatz M (2007). Stem cells from umbilical cord blood differentiate into myotubes and express dystrophin *in vitro *only after exposure to *in vivo *muscle environment. Biol Cell.

[B26] Gussoni E, Bennett RR, Muskiewicz KR, Meyerrose T, Nolta JA, Gilgoff I, Stein J, Chan YM, Lidov HG, Bönnemann CG, Von Moers A, Morris GE, Den Dunnen JT, Chamberlain JS, Kunkel LM, Weinberg K (2002). Long-term persistence of donor nuclei in a Duchenne muscular dystrophy patient receiving bone marrow transplantation. J Clin Invest.

[B27] Lakshmipathy U, Verfaille C (2005). Stem cell plasticity. Blood Rev.

[B28] Condorelli G, Borello U, De Angelis L, Latronico M, Sirabella D, Coletta M, Galli R, Balconi G, Follenzi A, Frati G, Cusella De Angelis MG, Gioglio L, Amuchastegui S, Adorini L, Naldini L, Vescovi A, Dejana E, Cossu G (2001). Cardiomyocyto induce endothelial cells to trans-differentiate into cardiac muscle: implications for myocardium regeneration. PNAS.

[B29] Terada N, Hamazaki T, Oka M, Hoki M, Mastalerz DM, Nakano Y, Meyer EM, Morel L, Petersen BE, Scott EW (2002). Bone marrow cells adopt the phenotype of other cells by spontaneous cell fusion. Nature.

[B30] Ying QL, Nichols J, Evans EP, Smith AG (2002). Changing potency by spontaneous fusion. Nature.

[B31] Wurmser AE, Gage FH (2002). Stem cells: cell fusion causes confusion. Nature.

[B32] Wang X, Willenbring H, Akkari Y, Torimaru Y, Foster M, Al-Dhalimy M, Lagasse E, Finegold M, Olson S, Grompe M (2003). Cell fusion is the principal source of bone-marrow-derived hepatocytes. Nature.

[B33] Sohn RL, Gussoni E (2004). Stem cell therapy for muscular dystrophy Expert Opin Biol Ther.

[B34] Ferrari G, Stornaiuolo A, Mavilio F (2001). Failure to correct murine muscular dystrophy. Nature.

[B35] Jackson KA, Snyder DS, Goodell MA (2004). Skeletal muscle fiber-specific green autofluorescence: potential for stem cell engraftment artifacts. Stem Cells.

[B36] Vieira NM, Brandalise V, Zucconi E, Jazedje T, Secco M, Nunes VA, Strauss BE, Vainzof M, Zatz M (2008). Human multipotent adipose-derived stem cells restore dystrophin expression of Duchenne skeletal-muscle cells *in vitro*. Biol Cell.

[B37] María LS, Rojas CV, Minguell JJ (2008). Signals from damaged but not undamaged skeletal muscle induce myogenic differentiation of rat bone-marrow-derived mesenchymal stem cells. Exp Cell Res.

[B38] Chen G, Quinn LS (1992). Partial characterization of skeletal myoblast mitogens in mouse crushed muscle extract. J Cell Physiol.

[B39] Chen G, Birnbaum RS, Yablonka Reuveni Z, Quinn LS (1994). Separation of mouse crushed muscle extract into distinct mitogenic activities by heparin affinity chromatography. J Cell Physiol.

[B40] Tatsumi R, Anderson JE, Nevoret CJ, Halevy O, Allen RE (1998). HGF/SF is present in normal adult skeletal muscle and is capable of activating satellite cells. Dev Biol.

[B41] Edmondson DG, Olson EN (1993). Helix-loop-helix proteins as regulators of muscle-specific transcription. J Biol Chem.

